# Pacemaker Lead Implantation Through an Unrecognized Right Subclavian Vein Occlusion: A Case Report

**DOI:** 10.7759/cureus.105236

**Published:** 2026-03-14

**Authors:** Akihiro Mizuno

**Affiliations:** 1 Department of Cardiology, Eiju General Hospital, Tokyo, JPN

**Keywords:** central venous obstruction, hemodialysis, pacemaker implantation, subclavian vein occlusion, transvenous lead placement

## Abstract

Central venous occlusion can pose significant challenges during transvenous pacemaker implantation. We report a case of successful pacemaker lead implantation through an unrecognized chronic right subclavian vein occlusion. A 77-year-old male on maintenance hemodialysis was referred for permanent pacemaker implantation due to symptomatic bradycardia. During the procedure, venous access was obtained via the right subclavian approach, and a guidewire was advanced toward the superior vena cava. Subsequent venography revealed chronic occlusion of the right subclavian vein with well-developed collateral circulation. Despite the occlusion, the guidewire traversed the obstructed segment smoothly, allowing successful advancement and positioning of the pacing lead in the right ventricle with stable electrical parameters. No vascular injury or immediate complications occurred. This case demonstrates that cautious guidewire traversal of chronic venous occlusion may allow standard transvenous lead implantation and help avoid more invasive alternatives in selected patients.

## Introduction

Central vein stenosis and occlusion are common in patients with prior hemodialysis catheterization or indwelling cardiac leads [1,2]. With the increasing prevalence of cardiac implantable electronic devices and long-term vascular access for hemodialysis, the incidence of central venous obstruction is rising and poses important procedural challenges during device implantation.

Lead-related venous obstruction may remain clinically silent because of collateral development [2]. When venous occlusion is encountered during pacemaker implantation, conventional management strategies include contralateral venous access, surgical epicardial lead placement, lead extraction to regain venous access, or endovascular recanalization techniques such as balloon venoplasty [3-7]. Occlusion of the proximal subclavian vein may significantly complicate pacemaker lead implantation and increase procedural complexity [4].In some patients, venous obstruction may remain unrecognized before the procedure because collateral venous circulation can partially compensate for the occluded segment. In such situations, guidewire advancement may initially appear feasible, potentially creating confusion during lead placement.

We present a case of successful transvenous pacemaker implantation through a chronically occluded right subclavian vein without planned extraction or formal recanalization. This case highlights an important procedural pitfall in which collateral venous pathways may mimic the main venous route, potentially leading to misinterpretation during guidewire or lead advancement. Recognition of this possibility is important for operators performing device implantation when venous obstruction is not suspected.

## Case presentation

A 77-year-old man with a history of hepatocellular carcinoma, prior cerebral infarction, diabetes mellitus, and end-stage renal disease on maintenance hemodialysis was admitted because of symptomatic bradycardia during hemodialysis. An arteriovenous fistula had been created in the left upper extremity in 2019. During a hemodialysis session, marked bradycardia was noted. Twelve-lead electrocardiography revealed a 2:1 atrioventricular block with a ventricular rate of approximately 40 beats per minute. He was transferred to our hospital for further evaluation and management.

On admission, blood pressure was 186/77 mmHg, heart rate 43 beats per minute, and oxygen saturation 100% on room air. No upper extremity edema or jugular venous distention was observed. Transthoracic echocardiography demonstrated a left ventricular ejection fraction of 35% with diffuse hypokinesis and an inferior vena cava diameter of 11 mm. Laboratory testing revealed anemia, elevated inflammatory markers, severe renal dysfunction, and markedly elevated B-type natriuretic peptide (Table 1).

**Table 1:** Laboratory data on admission Reference ranges represent institutional standards. LDH: lactate dehydrogenase; AST: aspartate aminotransferase; ALT: alanine transaminase; γ-GTP: gamma-glutamyl transferase; ALP: alkaline phosphatase; BUN: blood urea nitrogen; HbA1c: glycated hemoglobin; CK: creatine kinase; CK-MB: creatine kinase-MB; BNP: B-type natriuretic peptide

Parameter	Value	Reference range
WBC	4,600 /µL	3,300–8,600
Hemoglobin	12.3 g/dL	13–17
Platelets	315 ×10³/µL	150–350
Sodium	137 mEq/L	135–145
Potassium	4.9 mEq/L	3.5–5.0
Albumin	3.4 g/dL	3.8–5.3
LDH	237 IU/L	120–240
AST	21 IU/L	<40
ALT	8 IU/L	<40
γ-GTP	11 IU/L	<50
ALP	80 IU/L	38–113
BUN	22.0 mg/dL	8–20
Creatinine	3.91 mg/dL	0.6–1.2
HbA1c	5.80%	<6.0
CK	16 IU/L	50–200
CK-MB	1 IU/L	<5
BNP	4040.8 pg/mL	<100

A temporary transvenous pacemaker was placed via the right internal jugular vein on the day of admission. The pacing lead advanced smoothly without significant resistance, and ventricular pacing at 70 beats per minute was initiated. On hospital day 4, permanent pacemaker implantation was performed.

**Figure 1:**
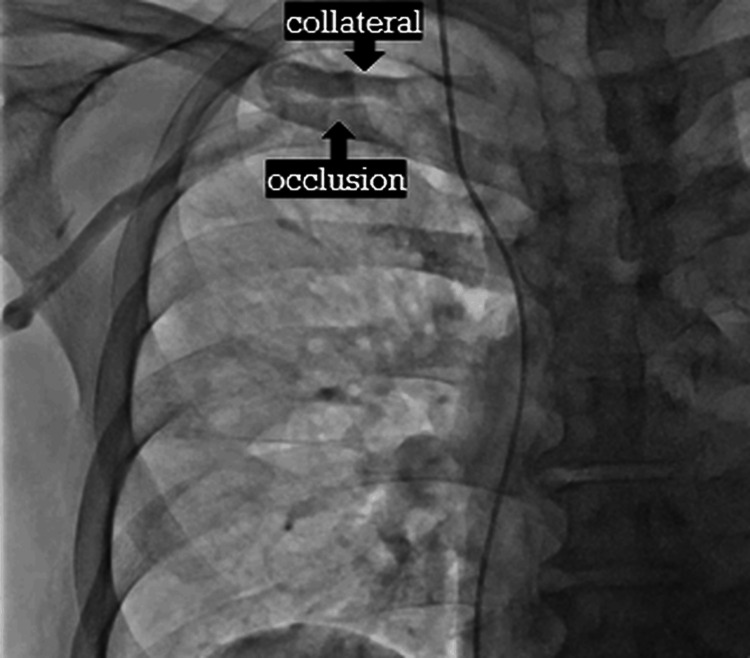
Pre-procedural venography Pre-procedural venography demonstrating chronic occlusion of the right subclavian vein with extensive collateral venous circulation. Arrows indicate the occluded segment and collateral pathways.

Because of the left upper extremity arteriovenous fistula, a right subclavian venous approach was selected. Pre-procedural venography demonstrated chronic occlusion of the right subclavian vein with prominent collateral circulation (Figure 1). The collateral vessel was initially mistaken for the main venous trunk.

During guidewire manipulation, the wire encountered transient resistance but was ultimately able to traverse the chronically occluded main trunk of the right subclavian vein. A 0.035-inch J-tip guidewire was used for venous access. After the guidewire successfully crossed the occluded segment, a peel-away sheath was advanced over the wire without significant resistance. A dual-chamber pacemaker was subsequently implanted. The pacemaker generator was an Amvia Sky DR-T (BIOTRONIK, Berlin, Germany). The atrial lead was a Solia S 45 (BIOTRONIK), and the ventricular lead was a Solia S 53 (BIOTRONIK). Both leads were successfully positioned with stable electrical parameters.

**Figure 2:**
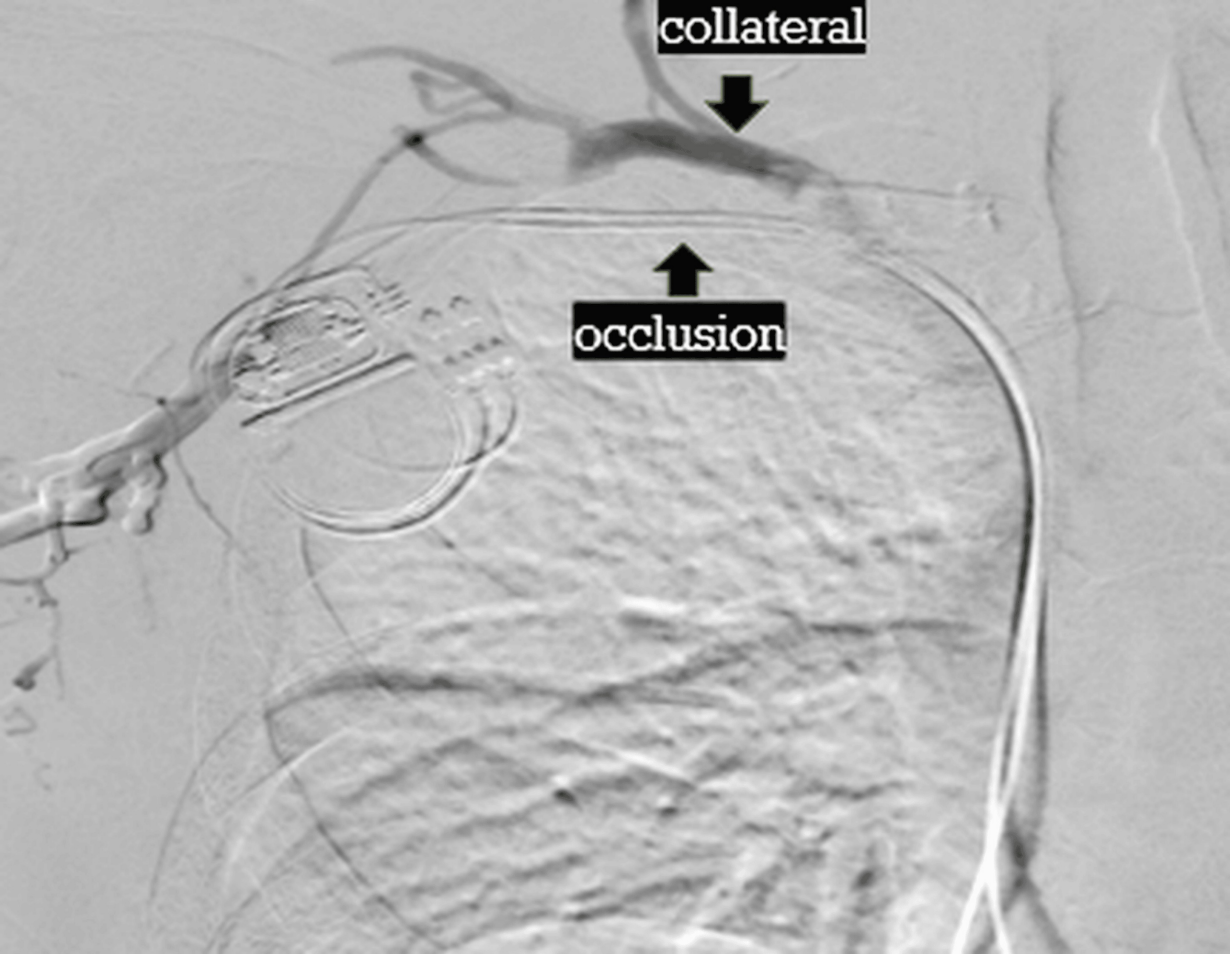
Post-procedural venography after pacemaker lead implantation Post-procedural venography after pacemaker implantation demonstrating two transvenous leads traversing the previously occluded right subclavian vein into the right atrium and right ventricle. No contrast extravasation or evidence of vascular injury is observed. The guidewire and lead trajectory across the occluded segment are visible.

Subsequently, a venous dissection at the site corresponding to the initial venography was suspected based on the lead position, and repeat venography was performed. Although it was recognized intraoperatively that the occluded segment had been crossed, lead position, sensing, and pacing thresholds were stable, and no contrast extravasation or evidence of vascular injury was observed (Figure 2). The procedure was therefore completed without additional intervention.

The postoperative course was uneventful. Post-implantation electrocardiography demonstrated stable ventricular pacing. During follow-up, no upper extremity swelling, venous congestion, or thrombotic complications were observed, and pacemaker function remained normal. The patient was discharged home on hospital day 11. No pacemaker-related complications occurred during follow-up. The patient died 11 months later due to the progression of hepatocellular carcinoma.

## Discussion

Central venous stenosis or occlusion is a well-recognized complication in patients undergoing repeated central venous catheterization or cardiac device implantation. Previous reports have described various techniques to overcome such venous obstruction, including hydrophilic guidewires, venoplasty, or alternative venous access routes. However, in many cases, the obstruction is identified before or during the procedure through venography or resistance to wire advancement. In the present case, the venous occlusion was not recognized prior to the procedure and was unexpectedly encountered during routine right subclavian venous access for pacemaker implantation. A standard guidewire was able to cross the occluded segment without the use of specialized recanalization techniques. This situation illustrates an important procedural scenario that may occur in clinical practice, particularly when venous obstruction is unsuspected. Another notable feature of this case was the presence of collateral venous pathways that initially appeared similar to the main venous route. Such collateral circulation may potentially mislead operators during lead advancement and create the impression of a patent central vein. Awareness of this possibility is important in order to avoid procedural confusion or complications during device implantation. Therefore, this case highlights a practical procedural pitfall during pacemaker implantation. Careful venographic evaluation and attention to unusual venous anatomy are essential when advancing guidewires or leads through the subclavian venous system. Central venous obstruction in hemodialysis patients often results from prior catheter placement and endothelial injury [1]. In patients with implanted cardiac devices, lead-related venous obstruction may remain clinically silent because of collateral development [2]. Management strategies include extraction to regain venous access [5], balloon venoplasty to restore patency [6], or advanced puncture techniques such as the balloon-target puncture method [7]. In congenital heart disease patients, complex occlusions, such as superior vena cava baffle obstruction after Senning repair, have also been successfully managed with transvenous lead placement [8]. In our case, inadvertent traversal of a chronically occluded subclavian vein allowed successful implantation without formal recanalization or extraction. This approach should be undertaken cautiously due to potential risks, including vascular perforation and thrombosis.

## Conclusions

We describe a case of successful transvenous pacemaker lead implantation through an unrecognized chronic occlusion of the right subclavian vein. Chronic venous occlusion may remain clinically silent due to collateral circulation and may only become apparent during device implantation procedures. This case highlights a potential procedural pitfall in which collateral venous pathways may mimic the main venous route and create confusion during guidewire advancement. Careful venographic assessment and attention to unusual venous anatomy are important when advancing guidewires or leads during pacemaker implantation.
